# Severe and Enduring Anorexia Nervosa: Enduring Wrong Assumptions?

**DOI:** 10.3389/fpsyt.2020.538997

**Published:** 2021-02-15

**Authors:** Emilio Gutiérrez, Olaia Carrera

**Affiliations:** ^1^Department of Clinical Psychology and Psychobiology, College of Psychology, University of Santiago, Santiago de Compostela, Spain; ^2^Venres Clínicos Unit, College of Psychology, University of Santiago, Santiago de Compostela, Spain

**Keywords:** acculturation, body image disturbances, fat phobia, invalidation, treatment efficacy

## Abstract

To the extent that severe and lasting anorexia nervosa (SE-AN) is defined in terms of refractoriness to the best treatments available, it is mandatory to scrutinize the proven effectiveness of the treatments offered to patients. The array of so-called current evidence-based treatments for anorexia nervosa (AN) encompasses the entire spectrum of treatments ranging from specialized brand-type treatments to new treatments adapted to the specific characteristics of people suffering from AN. However, after several randomized control trials, parity in efficacy is the characteristic among these treatments. To further complicate the landscape of effective treatments, this “tie score” extends to the treatment originally conceived as control conditions, or treatment as usual conditions. In retrospection, one can understand that treatments considered to be the best treatments available in the past were unaware of their possible iatrogenic effects. Obviously, the same can be said of the theoretical assumptions underpinning such treatments. In either case, if the definition of chronicity mentioned above is applied, it is clear that the responsibility for the chronicity of the disorder says more about the flagrant inefficacy of the treatments and the defective assumptions underpinning them, than the nature of the disorder itself. A historical analysis traces the emergence of the current concept of “typical” AN and Hilde Bruch's contribution to it. It is concluded that today's diagnostic criteria resulting from a long process of acculturation distort rather than capture the essence of the disorder, as well as marginalizing and invalidating patients' perspectives.

*It is the theory that describes what we can observe*.

Albert Einstein

## Introduction

The term *severe and enduring anorexia nervosa* (SE-AN) underscores that a substantial number of AN patients are refractory to currents treatments and become chronic (1). As defined by Strober (2), “*Chronicity is refractoriness of illness—permanence of the disease state in spite of repeated exposures to state-of-the-art therapy*.” (p. 487). However, as Wonderlich (3) has pointed out, “*There is no hard and fast algorithm, rule, or criteria for designating a patient as having a chronic eating disorder*” (p. 470), but the implicit message is that intractability is due to the inherent complexity of the essence of AN in these patients. The SE-AN label has been applied to patients with an active disease of 5–10 years in duration (4), meaning most adult patients belong to this category (5).

However, since the aims of any AN treatments are a logical corollary of the current understanding of the disorder, the repeated treatment failures prompt one to consider the possibility that the current conceptualization of AN may be misleading. Therefore, this paper neither intends to review the SE-AN condition, nor recent proposals for managing it (6, 7), but to question misleading assumptions regarding core symptoms capturing the essence of AN, as stated in the second part of the title. From this perspective SE-AN could be the final link of a chain of events that began with the appearance of the body image disturbances (BID) construct^1^. Furthermore, the rapid and widespread acceptance of BID in the 1960s as the cardinal symptom of typical AN has caused a hiatus with previous clinical descriptions of AN spanning almost a 100 years since the work of Marcé (10). Moreover, the BDI construct has created the fallacy that patients who do not spontaneously confirm it are suspected of being outliers or try to confuse doctors by hiding information, telling lies, and being manipulative. Third, there is some evidence that mental health professionals indoctrinate, acculturate, and export the BDI construct to apprehensive adolescents who have to deal with a serious disorder in their transition from adolescence to adulthood. Accordingly, the present work maintains that (a) the intractability of SE-AN ignores the contribution of the cumulative iatrogenic potential effects of successive failed treatments grounded on faulty assumptions and (b) the conception of SE-AN itself could be the result of cross-feedback between misleading assumptions and the increasing iatrogenic effects of failed treatments (11).

## The Creation of the Body Image Disturbances (BID) Myth

In his introduction to the Conference on Anorexia Nervosa and Related Disorders held in Swansea (Wales) in September of 1984, Gerald Russell remarked on the increase in the number of scientific publications on anorexia nervosa and bulimia in Medline^2^. Furthermore, Gerald Russell was concerned about the impact of this increased interest on the symptomatic clinical picture of anorexia nervosa, and stated: “*We should also remember that patients may tell us they started to induce vomiting when questioned by their doctors about this practice, or after reading a description in a magazine. This is a sobering thought which should increase our caution when we communicate our research findings, in case they are transmitted widely through the popular press and the media and reach susceptible individuals*” [(12), p. 107].

This call for professional responsibility highlights the configurative role of clinical and research activity on the psychopathological profile of AN patients. As Gerald Russell mentioned regarding BID: “*In recent years there has been a shift in emphasis on the nature of the central psychopathology of anorexia nervosa, with a greater stress on the patient's morbid preoccupation with her body weight and her dread of fatness. Hilde Bruch was probably first in 1962 to refer to a ‘disturbance of body image’ as part of a more general ‘perceptual and conceptual’ disturbance” (p. 103)*.

The appearance of the BID construct was a hiatus, rather than an evolution in the conceptualization of self-starvation. In the 1940s, due to the differentiation of AN from Simmond's disease (13–15), there was a return to the foreground of the relevance of psychological factors. This change in the emphasis on the nature of the central psychopathology of anorexia nervosa crystallized in the appearance of BID that is body and shape concerns and fear of fatness in patients with AN as the widespread explanation for their drive toward thinness. This new picture was the culmination of a trend initiated in the 1940s when, according to Casper (16), “*comments indicating concern about the shape of the body are rare in case reports before the forties, but then become the rule*” (p. 3).

This new endeavor was mainly colonized by the clinical descriptions of psychoanalysts after intense psychoanalytic treatment. This aspect is crucial since the golden rule of psychotherapy states that to work together, both patient and therapist must share a common language. Cases of AN are characterized by the stubbornness of teenagers in recognizing their illness, and adult therapists who impose their preferred specialized language on patients in line with their training. However, the therapist's language emanates from an underlying theory that, as Einstein said, not only tells the therapist what to observe^3^ but also tells patients the important aspects of their suffering. At variance with what happens in quantum physics, where measurements are no longer considered passive acts of observation but an active interference of the very essence of the object under study, tools for the diagnosis and treatment of mental disorders in general, and anorexia nervosa in particular, are awkward and unsophisticated probes that we stick into the human material. In much the same way as the very act of observation in physics interferes with the subatomic field, our attempts to evaluate symptoms are open to interference, acculturation, and even indoctrination.

As a result of psychoanalytic delving around the 1940s, the underlying motives of self-starvation began to emerge, as reported by Nicolle (18), who noted: “*In all cases I have seen, behind the trivial excuses offered lurks a fanatical desire to be thin and a dread of obesity. Miss M. said: ‘I am terrified of getting fatter or even of not getting thinner.’ The psycho-analytic interpretation of this state of mind would regard fatness as the sign of indulgence in the pleasures of the mouth and that the intense guilt associated with these impulses must be penalized by starvation and purgation. Such mechanisms can be brought to light in analytic investigation of these patients, but their restoration to consciousness does not seem to produce an amelioration of the condition as in other compulsive states*” (p. 158). A decade later aspects such as the “*fear of gaining weight and a horror to becoming fat*” [(19), p. 109] were more commonly reported.

Rahman et al. (20) were the first to connect conscious dieting with unconscious motives in their comments on 12 cases: “*the remarks about fatness were utilized as excuses, presumably to avoid touching on more painful topics…* [as intense analysis reveal that]… *In all our cases the reticence about sexual topics was noteworthy. There was no case in which a history of masturbation was elicited. Most of them resented questioning of any kind but especially concerning sex*” (pp. 354–355). This connection was announced in the commentary made by Patterson Brown to the Nicolle's (18) paper read before The Royal Society of Medicine on November 8, 1938, linking superficial dieting to its symbolic function by remarking on how Nicolle had “*related the not eating to a fear of growing fat, and consequently sexually unattractive to the male. This explanation is superficial and inadequate as it does not recognize the active repudiation of sexuality which is going on in these cases and which I should like to stress. At the deeper and more primitive level of the mind where this occurs the ingestion of food symbolizes impregnation and obesity pregnancy*” (p. 162)^4^. On these grounds, Waller et al. (21) launched their theory of the fantasies of oral impregnation^5^. These fantasies of impregnation were soon to be widely reported, for instance, Meyer and Weinroth (24), who were impressed “*by the coincidence of environmental pregnancies with modifications of the clinical history*” (p. 393).

The in-forming capacity of the deep analysis process is well-illustrated in the following paragraph taken from Loeb (23) who notes: “*Fantasy life largely revolved around sexuality, urination, and masturbation, the patient equating masturbation with guilt and dirtiness - masturbation causing a cancer - cancer representing a growth that kills - and a growth as a pregnancy, saying ‘people who are thin can't have babies’ and ‘I lost weight because my stomach looked so big, it looked like I was pregnant*” (p. 448). However, this colonization of the beliefs about oral impregnation fantasies has overlooked Nemiah's question of “*why, since oral impregnation fantasies are common, they so rarely lead to anorexia nervosa*” [(25), p. 263]. A similar inconsistency regarding the discrepancy between the high prevalence of diets and the low incidence of AN shall be examined below.

Nonetheless, it was precisely against this psychoanalytical emphasis on the symbolic significance of food, fears of oral impregnation, and incestuous involvement, and its usefulness for an effective treatment that motivated Bruch's seminal proposal of the core elements of AN. Bruch (26) had no qualms in stating this an instance of adultomorphizing of early behavior^6^ “*which needs to be understood in not for what purpose food is used in the psychic economy, but how it has become possible for a body function to be transformed in such a way that it can be misused in the service of non-nutritional needs*” (underlined text were italics in the original, p. 467). Much in the line with the double-bind theory of schizophrenia (27), Bruch focused the lens on the mother-child early interaction process resulting in an incorrect programming of patients' inner awareness during this interaction.

In a later contribution, Bruch (28) launched her conjectural triad of disordered psychological functioning, namely “*disturbance in body image of delusional proportion…disturbance in the accuracy of perception or cognitive interpretation of stimuli arising from the body… and a paralyzing sense of ineffectiveness*^7^… [which was based] *on the observations extending over 10 years of 12 patients (3 males and 9 females) whose conditions closely resembled the original image of anorexia nervosa*” [(28), pp. 187–191]. Despite the exiguous number of patients^8^, a fact rarely mentioned in the literature [i.e., (31)], Bruch's foresight remained unchanged in a posterior paper (30) where she addressed the differential diagnosis between genuine, pure, true, primary, classical, and typical AN^9^, in opposition to secondary, pseudo, non-specific, or atypical AN. In this paper (30), after recruiting 33 more patients, together with the 12 originally described in her previous paper, she performed a debugging task to differentiate the typical anorexia nervosa observed in 30 cases “*characterized by alterations in body image delusional proportions*,” from “*13 cases of refusal to eat in the service of neurotic or schizophrenic conflicts*” (p. 566). Later on, in her book *Eating Disorders: Obesity, Anorexia Nervosa, and the Person Within* (34), Bruch once more increased the sample to 70 patients (60 females and 10 males), 50 of whom were considered typical AN cases (45 females and 6 males), for whom she maintained the same triad of characteristics mentioned above.

Since its inception, Bruch's proposed key role of BID has been hailed to the Olympus of undisputed psychiatric wisdom *before* any empirical evidence gathering apart from Bruch's theoretical discovery (35). For example, as early as 1966 Daniel Cappon's letter (36) to the editor of the *Journal of the Canadian Medical Association* refuted Farquarson and Hyland's (37) 20 to 30-year follow-up report on 15 patients treated by these authors from 1932 to 1943. In Cappon's letter to the editor^10^, he asserted that Farquarson and Hyland's (37) paper was misleading for a number of reasons, among which was the lack of acknowledgment that “*The cardinal aspects of the syndrome are disturbances of body image and concept of physical self, failure to interpret enteroceptive signals, especially those of appetite (often due to a fear of opening the floodgates for eating, sex, etc., and non being able to stop), and denial of fatigue*” (p. 1,062). What triggered Cappon's commentary was Farquarson and Hyland's (37), Table 1 observation that teasing about being fat in adolescence cannot be taken as the sole origin of the abhorrence and fear of obesity. This same line of reasoning opposing the rarity of the AN, against the epidemic of diets among adolescent girls and young women was expressed more recently by Casper (42).

**Table 1:** Relevant opinions regarding being teased as precipitating factors of dieting and slimming practices in AN.

Langdon-Brown (38)	*“Anorexia was rationalized by one of three grounds: (1) humanitarian pleas; (2) slimming, especially if there had been teasing because the young woman had been getting somewhat plump; (4) on religious grounds” (p. 308)*.
Ryle (13)	*“Slimming', usually instigated by the ridicule of school friends on account of adolescent plumpness, is generally considered second in importance to these emotional crises. Four cases at least in this group originated in this way” (p. 895)*.
Ryle (39)	*“Of initiating or contributory factors, other than slimming in response to teasing on the score of adolescent plumpness (and this can rarely be accepted as a sole cause), love affairs, broken engagements, school attachments and jealousies, home sickness (especially in the case of girls sent abroad), unhappy home life, spoiling by devoted parents, mental shocks, examinations and overwork, and convalescence from a physical illness or operation are all noteworthy” (p. 26)*.
Berkman (40)	*“of young girls, 13 to 15 years of age, who during the grade school years…they may realize that they are overweight or may be cruelly reminded of this fact by others. As a result they markedly cut down on their food intake” (p. 682)*.
Selvini Palazzoli (32)	*“But to these predisposing generic pathogens we can also add more specific ones: the fashion of being thin and sophisticated, the widespread commercials on diets and drugs to lose weight, the continuous talks on calories and weight loss among family members and friends and, especially, the jokes reserved to women of the type in Rubens's paintings. Nowadays, our cultural environment does not accept the fat woman, doomed to be alone and rejected”* [(32)*, p. 58, translated from the original in Italian*].
Farquarson and Hyland (37)	*“It is extremely difficult to learn what the most important precipitating factors in anorexia nervosa may be, for one cannot fathom an adolescent's mind. Abhorrence and fear of obesity loom large in the thinking of many patients. In eight of our patients the onset took place when the patient was subjected to teasing about being fat, or was simply overly conscious of obesity. One cannot accept this as the sole factor, however; otherwise anorexia nervosa would be a common and not a rare disease” (p. 419)*.
Stice et al. (41)	*“One striking finding is that low BMI, low dieting, negative affect, and functional impairment predicted onset of AN and a low BMI, whereas the risk factors relating to cultural pressures for thinness and resulting body dissatisfaction and weight control behaviors did not… The fact that thin ideal internalization and body dissatisfaction did not predict AN implies that cultural pressure for thinness do not increase risk for AN” (p. 50)*.

The caution against excessive generalization referred to by Farquarson and Hyland (37) had already been voiced 30 years earlier by Ryle [(13), see Table 1]. In the eight vignettes included in Ryle's (13) paper, five correspond to the youth group (composed of 33 patients, mean age 20 years), and in only one case, case 5, a girl aged 19 “*began to ‘bant,’ having been told that she was too fat*” (p. 896). In a later paper, Ryle [(39), see Table 1] was even more explicit about the error in considering dieting and slimming practices in response to teasing about fat as the sole cause^11^. Furthermore, Berkman [(40), see Table 1] highlighted the link teasing-slimming was restricted to a subgroup of young girls (13- to 15-year-olds). Similarly, according to Langdon-Brown [(38), see Table 1], slimming was only one of the three reasons upon which patients rationalized their restricted eating. Finally, dieting because “*abhorred fat people or the idea of becoming fat*” was mentioned in less than a third of cases reported by Kay and Leigh (44).

Dieting, however, was soon considered the main road for the relentless pursuit of thinness. What had changed? Obviously, as Bruch pointed out the slimness, conscious society had contributed to a new risk factor: the ubiquitous thin ideal cliché, via its internalization by the pre-anorectic adolescent patient. Actually, it was not Hilde Bruch, but Selvini Palazzoli [(32), see Table 1] who first mentioned the cultural ideal of slimness as a factor in the etiology of AN^12^.

Nevertheless, the mantra linking social pressure to fit the ideal thinness, and its internalization by vulnerable adolescents, became far more widely accepted than supported either by research or by patients themselves. The assumption that thin internalization is a risk factor in AN has been rebutted by the most extensive study to date involving data from 3 prevention trials involving High-Risk Adolescent Females [(41), see Table 1]. Likewise, Striegel-Moore and Bulik (45) lucidly mention that the thin-ideal internalization framework resides more in the minds of clinicians and researchers than in the minds of patients. The idea that media influence is a direct causal agent is so widespread that, as Polivy and Herman (46) pointed out, it would be necessary to explain why the incidence of AN does not have pandemic proportions.

### A Brief Overview of Research on the BID Construct

Since the publication of the *DSM-III* (47), the criteria for AN diagnosis have experienced an uneven weakening of signs and symptoms. Thus, in the *DSM-5* (48) two of the three criteria for AN diagnosis involve unobservable symptom complexes, i.e., body image disturbance and fear of fatness. Moreover, the removal of the amenorrhea criterion, the only sign (criterion A) referring to low bodyweight, has progressively become less stringent compared to the demanding clear cut-off point of at least 25% body weight loss in the former *DSM-III*.

Whilst the list of signs has undergone profound changes, in comparison the symptoms that are part of the BDI construct have remained relatively stable. However, despite its status as a core symptom for AN diagnosis, the BDI construct has not obtained sufficient empirical support to dispel the doubts of being an epiphenomenon of the characteristic low weight of these patients. This perspective was already clearly pointed out by Berkman (40), even before the publication of the cognitive, emotional, and behavioral changes of starvation arising from the Minnesota starvation study (49, 50)^13^, also mentioned by Dubois (19). According to this view, both somatic symptoms as well as the mental make-up of AN patients would be deemed consequences of starvation.

Nonetheless, the continued presence of symptoms of the BDI construct in the latest *DSM-V* and *ICD-11* (51) would suggest that the accumulated evidence around this construct is irrefutable, which is not so. In fact the evidence on the BDI construct around the time the *DSM-III* was issued (52) was less than promising with a “*marked inconsistency of findings*…[and] *a fairly consistent finding across studies is greater intersubject variability*” [(53), p. 493]. In the same vein Garner and Garfinkel (54) noted, “*It is unclear whether body image disturbances are pathogenic or strictly a byproduct of a serious eating disorder*” (p. 280).

Although currently BDI is mostly considered a maintenance factor, originally the conception of BDI was the product of an informal fallacy derived from a reasoning error known as the “*post-hoc ergo propter hoc*” fallacy, also known as “*post hoc” fallacy*. This fallacy asserts that if one event occurs after another, the second event is a consequence of the first. In other words, in the acute phase of AN, the “douce indifference” (55) of severely emaciated AN patients shows no concern for their physical appearance, or even claiming they want to stay as they are, or even lose a little more weight, then this apprehensive behavior is the cause of their emaciated state. However, since its discovery by Hilde Bruch likely correlation has been misinterpreted as causation. This flawed form of reasoning was common at the time of the “double bind” hypothesis of schizophrenia (27) that inspired both Bruch (34) and to a greater extent Selvini Palazzoli (32).

The BID construct has two main unsolved problems. The first of them has to do with its multifaceted nature, while the second problem area has to do with its clinical management. The first dissection of the BID construct into its main components was made by Garner and Garfinkel (54). According to these authors, the BDI construct would encompass three types of disorders: perceptual, cognitive, and affective. The first type of disorder has generated extensive research framed under the label of body size distortion, while the study of the last two aspects has been addressed as body dissatisfaction (53). However, in addition to research on the perceptual and cognitive-affective components of the BID construct, Legenbauer et al. (56) remark insufficient attention to other components of BID construct such as dysfunctional body-related behaviors, such as body checking and avoidance behavior. Nevertheless, more than 30 years later, some authors consider BID construct as one of “*the most discussed and controversial symptoms in AN*” [(57), p. 42], and that the “*distinctive features and mechanisms of BID remain unclear, specifically in regards to the contributions of sensory perceptual distortions v. cognitive–affective disturbance*” [(58), p. 642].

Undoubtedly, because BID was originally conceived as a sensory-perceptual deficiency (59) this perceptual aspect, that is, the tendency of patients with AN to overestimate their body size, has been the component of the BID that concentrates greater research efforts. However, this aspect encompasses such a diversity of methodologies (from movable calipers to eye tracking techniques) and stimuli (own body to 3D avatars) and different control groups that a systematic review exceeds the limits of this paper. In addition, to research on the perceptual and cognitive-affective components of the BID construct, Legenbauer et al. (56) remark insufficient attention to other components of BID construct such as dysfunctional body-related behaviors, such as body checking and avoidance behavior. Still, with respect to research of the cognitive-affective component of BID construct, as Eshkevari et al. (60) warn, patients' exteroception has been prioritized in BID research in detriment of BID interoceptive aspects that are an essential source of information for the AN patients' experience of their bodies. However, as is often the case when research activity on a phenomenon is intense, this intensity is often misinterpreted as evidence of proven results. In this regard, as Smeets (61) noted: “*In research on distorted body image, many assumptions have been made. As is often the case with assumptions, they have come to have a life of their own and, over time, have acquired the status of established facts*” (p. 76).

On the other hand, a second controversial issue is related with the need to address BDI in the treatment of AN. At this respect Bruch (34) stated that correction of the BDI was a “*precondition for recovery*” (p. 90), but evidence accumulated to date is far from clear in this regard. It is almost a convention in any review of the BID literature to mention that the severity of BID predicts long-term outcome in AN patients, and that persistence of BID predicts the rate of relapse (62). However, there is a blatant disproportion between the frequency of these claims and the supporting evidence and most importantly the continuity of the “uniformity myth,” which erroneously considers ED as a homogeneous category where the differences between the different disorders are less important than their communalities, as proposed by transdiagnostic theory (62).

Relevant information about the pertinence of targeting BID construct and its relation with treatment outcomes in AN is found in recent work from an Italian team researching focus on body-image concern (BIC), a core construct both in CBT for adults and in CBT-E—an enhanced form of CBT (63) based on the transdiagnostic theory (64). BIC entails three components, namely “Preoccupation with shape/weight,” “Fear of weight gain,” and “Feeling fat.” However, Calugi and Dalle Grave [(65), p. 584] found that “*our data suggest no relationship between the change of BIC components that occurs during treatment and the change in BMI centile over time*.” Notwithstanding this shortcoming the authors did not moderate the importance of the core BID construct but they stress that CBT “*works as a whole; in other words, the improvement in body weight seems to be mediated by its overall application rather than its single components*.” In a posterior paper Calugi et al. (66) acknowledge that despite CBT-E directly addresses BIC, they concluded that “*the research done to date has not yet clarified whether body-image concern is in fact a core characteristic of eating-disorder psychopathology or merely an epiphenomenon*” (p. 64).

Also relevant to this matter are secondary analyses conducted by members of the Anorexia Nervosa Treatment Outpatient Study (ANTOP). ANTOP is the worldwide largest randomized controlled trial and compared focal psychodynamic therapy (FPT), enhanced cognitive behavioral therapy (CBT-E), and optimized treatment as usual (TAU-O) in adult outpatients with AN (67). These authors documented what they denominate a “*persistency effect*” of BDI remaining high while body weight increased, irrespective of treatment arm (68). Furthermore, they reported that “*The associations of body image perceptions with symptoms of depression and anxiety appeared to increase along the course of treatment despite overall improvement of depression, anxiety, and body image disturbances with treatment duration*” [(69), p. 146]. The apparently contradictory results are commented by the authors in a later publication where body image perceptions predict symptoms of depression and anxiety in the course of outpatient treatment “*which in turn predicts depressive symptoms at the end of therapy which in turn predicts the outcomes body mass index and EDI-2 sum score at 12 months follow-up*” [(68), p. 49]. However, although these authors speculate about the possibility that the relationship of body image self-appraisal and symptoms of depression might be bidirectional, they remark the importance of addressing BDI during AN treatment. However, they caution about probable “*adverse effects of eventually increased stress levels in patients with AN when explicitly focusing on body image self-appraisal during psychotherapeutic intervention…[which] subsequently leads to worsening of affective comorbidities (and not to the intended habituation) and hence to less favorable treatment outcomes*” (p. 56). Still, a recent meta-analysis of randomized controlled trials of body image interventions designed to target body image dissatisfaction among non-clinical populations, including fitness training, self-esteem enhancement, media literacy, and psychoeducation, found “*only a small effect size for improving body image across these interventions*” [(70), p. 163].

Nowadays, a panorama of mixed findings summarizes the state of the art regarding body image-related interventions covering most widely adopted strategies (cognitive restructuring, mirror exposure, video-confrontation, and virtual reality) but without even passing the promising results phase and the absence of replication being a main problem. However, in spite that the two main problem of BDI are unresolved and without promising prospects in the near future still there are proposals for re-classifying AN “*under a new category of body image disorders, together with other mental illnesses in which body image is the dominant feature, such as BN, body dysmorphic disorder (BDD), and muscle dysmorphia (MD)*” [(71), p. 14]. However, with respect to BDI “*We are still living mainly on assumptions*.”

A further aspect of the tyranny of the BID construct that should not be overlooked is the bias in the understanding of hyperactivity in AN. Mainstream thinking considers hyperactivity as a mere weight-losing strategy, that is, patients feel fat and want to lose weight because their BID is fuelled by social pressure to be thin. Hyperactivity has been described in anorexia nervosa by clinicians and researchers since the first modern descriptions of the illness (42, 72). Excessive exercise is a characteristic sign in AN with some estimates raising its prevalence as high as 80% (73). Although excessive activity was considered a fundamental clinical feature (74) before the *DSM-III* was released (52), hyperactivity has been traditionally considered a Cinderella among AN signs (75), and accordingly it has been assigned a secondary rank in the *DSM* series, and is conceptualized as a mere calorie burning strategy (76). This conceptualization contradicts research showing that excessive activity often precedes the onset of the disorder (77), as first pointed out by Janet (78) who stated that “*the exaggeration of the movement is sometimes anterior to the refusal of food and therefore precedes all these reasonings*” (p. 501). In the first case of AN described in detail in the literature, Lasègue (79) stressed that in AN the abstinence from food “*tends to increase the aptitude for movement*” (p. 266), and also early in the literature Janet acknowledged the negative impact of excessive activity upon eating when he stated that “*The exaltation of the strength, the feeling of euphoria, as it is known in the ecstatic saints, for instance, does away with the need of eating*” [(80), p. 242]. It was Gull (81) who was the first to acknowledge that this hyperactive profile should “*be controlled, but this is often difficult*” (p. 25), an observation that is valid for current treatments that consider excessive activity in AN as a voluntary calorie burning strategy at the service, of course, of the BDI construct. This interpretation has been recently challenged in a study (82) where the physical activity of AN patients was significantly modulated by environmental temperature beyond the eventual regulatory function of anxiety, negative affect, body dissatisfaction, and drive for thinness. It is worth noting that this modulation of environmental temperature is similar to the experimental evidence gathered from research with an analogous animal model of AN known as Activity-Based Anorexia (ABA) where ambient temperature (AT) is a critical factor contributing to the expression of excessive running activity. The increase in AT to 32°C completely prevented and fully reversed excessive activity in ABA rats (83, 84). Thus, incorporating warming as an adjunctive treatment for AN patients is a promising alternative to controlling hyperactivity (85, 86), and a heated environment (again at 32°C) reduced post-meal anxiety in AN patients (87).

## Typical an and Atypical an: Not Atypical an, but the Typical Doctor

Though numerous clinicians and researchers since Déjerine and Glauckler (43) have attempted to define AN as a separate nosological entity [i.e., (31, 32, 88–91)], none has had the significance of Hilde Bruch's seminal work. Thus, from the 1960s onwards primary anorexia nervosa was defined in terms of the pathological pursuit of thinness and its association to the disturbed body image (92). The binding association between BID and the typical presentation of AN implicitly entailed all other patients not presenting this characteristic were excluded and assigned to a dubious class of atypical AN. An unintended consequence of Hilde Bruch's use of the term “classical” as a synonym for “typical AN” was the usurpation of this qualifier for all the cases described from the time of Marcé (10), Lasègue (79), and Gull (81). Furthermore, the use of the term “classical” as synonymous to “typical AN,” which according to Bruch (28) “*closely resembled the original image of anorexia nervosa*” (p. 187), implied that the triad of characteristics were intrinsic to the very essence of AN. However, given that AN reports in the 60 years prior to Bruch's description, with the exception of the abovementioned antecedents that emerged from the psychoanalytic circle, were mostly devoid of any BID features, we are compelled to examine other alternative explanations. Among these possibilities the most accepted is the one that supports the absence of BID in the psychopathology of AN before Bruch (28), claiming that BID emergence is an evidence of the changing nature of AN. Thus, if the phenomenology of AN had changed, this change would exempt renowned doctors such as Marcé, Lasègue, Gull, Janet, Déjerine, among others, for being blind to Bruch's observations. Russell addressed this point when he said that “*neither Lasègue nor Gull drew attention to the psychological disturbances which appeared so striking lo late observers: the patient's disturbed experience of her own body* (30)*, ‘weight phobia’* (93), or ‘*morbid fear of fatness’* [(94, 95), p. 7]^14^.” Russell (12) had previously found that “*these simple observations have only been made in the past fifteen years or so. At the time they were hailed as new discoveries. Yet it would seem surprising that generations of able clinicians should have missed basic and plainly discernible features of the psychopathology*” (pp. 104–105). The explanation put forward by Russell to settle the matter was to state “*psychopathology of anorexia nervosa has changed between 1870s and the 1960s*” [(95), p. 7].

An alternative to the idea of a change in the phenomenology of AN claims that BIDs have always existed, but the absence of BID reports in the medical literature before Hilde Bruch can be explained by the reticence on the part of both patients and doctors. According to the main exponent of this view, Habermas (96), the scarcity of BID reports in the medical literature was due to “*the anorexic's tendency to hide their weight-related motivation, as well as to the corresponding unawareness of most physicians*” (p. 360). But, how could this epidemic of blindness among physicians be overlooked in the face of the obvious? The explanation provided by Habermas (97) illustrates well the role of “*pre-established expectations about what is observed and what is neglected*” by doctors themselves. Therefore, according to this point of view, the selective report and the physicians' BID rely more on membership to “schools of convictions” (98) and the physician's specific training, than on the changing nature of AN. As evidence of his thesis, Habermas (97) mentioned some exceptions to this ignorance in recording the intense fear of being overweight in six of the nineteenth-century case reports and that “*all but one of the authors were French*” and “*most of them were in some way related to the Salpétrière, were Charcot was working and lecturing*” (p. 263). Further on, Habermas adds: “*Once introduced into French psychiatric thinking, other clinicians also discovered the anorexic's fear of obesity, whereas the British and German doctors, not having their attention focused on weight-related fears by a national authority, simply did not see it*” (p. 269)^15^.

However, the fact most of these French authors reporting fears related to weight were linked to the Pitié-Salpêtrière Hospital in Paris, where Charcot had worked for 33 years (1862–1893), only implies they were under the influence of Charcot, and not that Charcot was right. Actually, one of Charcot's eminent students, Pierre Janet, criticized him for exaggerating on this matter: “*The authors who have observed such ideas seem to me to be inclined to exaggerate their importance. This is what certainly happened to Charcot, who used to seek everywhere for his rose-colored ribbon and the ideas of obesity*” [(80), pp. 234–235]^16^. Nevertheless, Habermas (100) recognized this prescriptive influence of Charcot's observation when he stated: “*This indicates that once an authority had established the legitimate expectation that weight phobia could or even should be found and described in anorexic patients this trait was much more readily identified and reported*” (p. 325). The expression “should be found” goes beyond merely inviting to search and requires a mandatory confirmation. Therefore, the typical and atypical categories are a creation of the typical doctor equipped with a set of fixed expectations and assumptions. This is not only about gathering the fruits of nature, but also sowing them.

Even if Russell's suggestion that the phenomenology of AN had changed were endorsed, the BID requirement for the diagnosis of typical AN still posed a further problem for patients who failed to neatly fit the clinical profile of Bruch's description of typical AN as they were thrown into the catch-all category of an atypical eating disorder in the first *DSM III* (52), “*a residual category for eating disorders that cannot be properly classified in any of the previous categories*” (p. 69).

Soon after the appearance of the *DSM-III* in 1980, the first case reports began to appear that did not meet the newly released *DSM-III* criteria for AN diagnosis, either because they did not meet the weight criteria (101), or the BID criteria (102, 103). Moreover, the role of BID in AN diagnosis was further called into question following the first epidemiological and clinical reports of atypical patients appearing in the Hong Kong area (104), who did not systematically express fears of gaining weight, hence the name non-fat phobic AN patients (NFP-AN). Thus, both weight concerns (105, 106) and body image disturbances (103, 107), two criteria that remain essential elements for the diagnosis of AN under the *DSM-5*, were not endorsed. The first reports from Hong Kong were soon followed by others from other non-Western populations such as Singapore, Malaysia, Ghana, India, Sri Lanka, and Japan, as well as Asian and South Asian patients living in Western countries (108). However, the incidence of these atypical cases was not restricted exclusively to Asian or African populations since the incidence of this type of patient had been estimated to be around 20% of patients treated in Western countries (109). This percentage of up to 20% of NFP-AN cases in Western countries, more specifically in the United States and Europe, is an important nuance when employing Western as a label opposed to East Asian societies. This geographical dichotomy proves to be inappropriate since in countries such as Japan FP-AN cases are more common than NFP-AN cases (110), and there are also high levels of body dissatisfaction and thin ideal internalization, particularly among the population of high school and college-aged women (111).

The first study exploring differences between typical AN, according to *DSM-III*, and atypical patients without the pillars of the BID construct, namely weight phobia and body image disturbance (33), concluded that the classification of patients into typical vs. atypical diagnostic subtypes was “*nosologically valid and clinically useful*” (p. 135). From this study onwards, a second wave of studies explored quantitative differences between NFP-AN and FP-AN patients according to the different cut-off points on the Drive for Thinness Scale in successive versions of the Eating Disorders Inventory [EDI, (112)]. Although the present paper does not seek to undertake an exhaustive in-depth review of existing studies, the results are inconsistent regarding differences in premorbid characteristics, both in eating disorders and in the general psychopathology and outcome of atypical NFP vs. FP typical AN patients^17^. Nonetheless, the overall tendency appears to be less eating-related pathology, less severe psychopathology, and a more favorable course for NFP-AN patients (33, 109, 110, 113–118).

However, the very existence of atypical NFP/AN patients has implications that go far beyond the differences from their typical AN counterparts. First, BID symptoms, supposedly pathognomonic symptoms of AN, seem to be culturally mediated and, in this sense, the current “typical *DSM* AN” is neither purer nor truer than other atypical presentations reported in non-Western and Western cultures. This point has been well-expounded in Steiger's (119) comments on Lee et al.'s study (120), stating that “*Diagnostic conventions dictate that such cases [NFP-AN] should be labeled ‘atypical’, but this may simply be an invention. Actually, the syndromes characterized by self-imposed wasting can exist quite ubiquitously, although the concept of 'fat phobia' fits many of them bad … anorexia nervosa has a much weaker connection with a Western 'culture of thinness' than previously thought … Western culture can motivate elaborations in which fatness concerns are characteristic, but such expressions do not need to represent defining examples of the syndrome*” (pp. 66–67).

Therefore, according to (120), instead of the “typical AN” designation, the term “conventional AN” would be more appropriate, since it emphasizes that it is the result of a procedure accepted as true or correct by convention. Accordingly, the very essence of AN would be not reflected in what Bruch signaled as prototypic, genuine, pure, classic, primary, or typical, but rather the so-called typical AN picture, as depicted since the *DSM-III*, would be the result of a slow and constant acculturation process spread through the therapeutic milieu and mass media (121). The point is not whether AN can exist without cultural contamination, but rather if there is excessive cultural contamination that is currently sanctioned by the *DSM*, and widely circulated by the mass media. This excessive cultural contamination maintains an unintentional process of indoctrination that formats the patients' experiences and invalidates their own personal experiences. Hence, the successive *DSM*s since the third version could be considered the most active agent in homogenizing what should qualify as typical AN. Probably, as Russell (12) mentioned, the phenomenology of AN has changed, but this change was initiated and maintained by mental health professionals who disseminate their expectations among families, patients, public in general, and among other colleagues during conferences, specialist training process, and so on. Obviously, this approach reverses the taken for granted conception that atypical presentations are intrinsically deviations while the current typical AN is natural. Consequently, clinicians and researchers are not passive observers of how culture modulates and colors patients' testimony about their motives for food restriction. On the contrary, they are active agents who handle the brush and the color palette and whose workings, disclosed both through the scientific literature or divulged to non-scientific audiences, are collected and disseminated by the mass media exercising a demand-conforming offer, as Russell (12) has cautioned^18^.

One of the aspects supporting the view that atypical AN is the true authentic AN without cultural contamination is the consistency of the clinical picture of AN between those patients described before 1960 and today NFP-AN patients. This means that for almost 100 years since first description by Marcé (10) to the seminal BDI publication by Bruch (28) what is considered today as atypical AN was actually the normal presentation as self-starvation was characterized by the absence of elements of the BID construct as motivating factors. Moreover, as above mentioned the absence of the fear of fatness and body image disturbances are characteristic in patients with NFP-AN in both non-Western countries, and around 20% of AN patients in Western countries.

Twenty-five years ago Russell (95) already expressed his caveat concerning the alleged core role of one of the elements of the BID construct in AN diagnosis by pointing out that “*The dread of fatness is likely to be a modern development in the psychopathology of anorexia nervosa. It need not persist in future generations of anorexic patients. The time may be approaching when it will be advisable to retreat from our cherished diagnostic criteria of anorexia nervosa, as there may be a false precision in the current formulation*” (p. 10). Nonetheless, the most widespread interpretation of the absence of BID in atypical AN is patient denial.

## Denial and Invalidation: The Relentless Pursuit of Fat Phobia

This section refers to the quasi-compulsive need of confirming that patients with AN suffer from fat phobia (a component of BID as explained in footnote^1^). Germane to this, *DSM-5* criterion C has lowered the *DSM-IV* charge of active “*denial of the seriousness of the current low body weight*”^19^ by a supposedly more descriptive “*or persistent lack of recognition of the seriousness of the current low body weight*^20^.” Despite this subtle change, it is doubtful that the clinicians' lenses are so polished as to discern the subtlety distinguishing active denial from persistent lack of recognition. From the patient's point of view, the transition goes from the experience of being accused of denying, to not wanting to recognize something that the clinician knows is there.

The notion that patients are manipulative, lie, and hide information is certainly old in the literature, but appears precisely when AN patients, previously rescued from the lures of endocrinology (13), were again rescued from the lures of psychoanalytic unconscious motivations. As mentioned above, this release was the merit of two psychoanalysts, Hilde Bruch and Selvini Palazzoli, but the ransom price was the charge of denial and untrustworthiness. Thus; Selvini Palazzoli (32) stated of AN patients: “*They always have excuses ready, they can lie to the point of ridicule*” (p. 27, translated from the original in Italian). Even Selvini-Palazzoli exonerated Gull and Lasègue^21^ from not being able to go further in their early description of AN as “*the patients' negative attitude prevented them from communicating their inner experiences to the physician and obstructed all attempts to delve into their psychological motives, the more so as most of them showed a marked lack of introspective powers*” [(32), p. 9]. This is in stark contrast to Lasègue's (79) statement that he was unable to obtain the reasons underlying food avoidance in the patients he consulted as “*None provided me in this retrospective investigation with information other than what I have reported: ‘I could not, it was too strong for me, and besides I was well'*” (p. 402, translated from the original in French). The same frustrating experience was reported by Berkman (124) who reported “*On being questioned, most of the patients are unable to express an opinion, or will not do so*” (p. 412).

What numerous clinicians have heard from AN patients has been an unremitting complaint regarding abdominal pain and discomfort: “*dans des douleurs gastriques*” [(79), p. 386]; “*to be troubled with indigestion*” [(125), p. 613]; “*epigastric distress*” [(126), p. 1,085]; “*organic disease of the stomach (ulcer)*” [(127), p. 745]; “*varied gastrointestinal symptoms*” [(128), p. 817]; “*indefinite gastrointestinal disturbance*” [(40), p. 681]; “*abdominal discomfort*” [(19), p. 113]; “*dull or burning pain in the epigastrium*” [(25), p. 256]; “*abdominal discomfort, a common cause of complaint in the early stages of treatment*” [(129), p. 1,771]; “*constipation and minor abdominal disconfort*” [(130), p. 345], “*abdominal tenderness […] and complaints of unbearable abdominal fullness follow the ingestion of even small amounts of food*” [(31), pp. 445–446], “*abdominal pain*” [(131), p. 438], and “*epigastric discomfort*” [(132), p. 594].

Interestingly, the same gastrointestinal complaints have been consistently mentioned by atypical AN patients (94, 101, 103, 108–110, 113, 118, 133). Likewise, a recent study (133) with typical AN patients reported, contrary to expectations, more gastrointestinal symptoms on a Gastrointestinal Dysfunction Scale than a comparison group consisting of NFP-AN patients. Thus, it is plausible to suggest that the absence of reports of gastrointestinal complaints in typical FP-AN patients in the literature was either the result of biased reporting by patients or selectively focused clinical questioning in accordance with diagnostic requirements. This unexpected finding reported by Lee et al. (133) is most interesting as gastrointestinal complaints has been recently identified as a predictor of positive outcome in a 30-year outcome report of the Gothenburg anorexia nervosa study (134).

Nevertheless, digestive discomfort is often seen as an excuse for hiding fear of fatness. This is another example of invalidation of the patient's own experience despite the fact that up to 16% of AN patients have gastric ulcers (135). Perhaps the phenomenology of BDI has changed due to cultural pressure, but this pressure does not seem to have influenced the uninterrupted report of digestive discomfort as an explanation for restrictive eating patterns from the time of Lasegue to recent reports of patients with atypical AN. This continuum deserves to be considered in good faith and not as active concealment and deception. Nonetheless, both past and present interpretations of the absence of BID reports by AN patients still continue to be attributed either to their marked lack of introspective powers (32), or to their defensive reaction, as Crisp (136) claimed: “*Individuals with anorexia nervosa deny their weight phobia, since general awareness of it would lead to their being considered by others as more responsible for their condition than is usually the case and would render them more vulnerable to outside influence by inviting much closer scrutiny of their motives and habits. Thus only a few anorectics are referred with an explicit history of postpubertal concern about their volume and shape, their dieting and weight phobia. The presence of weight phobia or shape or volume phobia and the overriding terror of fatness, within the context of low body weight and its attendant features described above, is pathognomonic of anorexia nervosa*” (p. 687).

However, to unveil these alleged pathognomonic symptoms referred to by Crisp requires substantial collaboration from patients, which leads to the question as to how this task is accomplished. First, during the clinical examination the clinician could attempt resolutely to discover the BID that fueled the patients' relentless pursuit of thinness. Kay and Leigh (44) already stressed how fundamental the psychiatrist's insistence was during the clinical examination when they asserted that “*A history of ‘voluntary dieting’ probably depends on the intelligence of the patient, her ability to rationalize, and her willingness to discuss her symptoms, no less than on the psychiatrists' zeal in searching out ‘motives’*”(p. 413).

Furthermore, in addition, patients are usually required to complete a certain number of paper-and-pencil self-reports in face-to-face interviews where patients are again invited to reveal in writing their beliefs and attitudes (is it that they weren't pathognomonic?), these patients are not prone to disclosing their inner thoughts either verbally or non-verbally.

Nevertheless, if the previous strategies proved to be unsuccessful there was still the option of psychotherapy. These strategies involved reversing the burden of proof where it was almost impossible for patients to prove their innocence under the ubiquitous suspicion of denial.

## The Role of the Bid Construct in the Acculturation and Indoctrination of Anorexia Nervosa

Undoubtedly, it is during treatment, especially when it involves hospitalization in eating disorder clinics, where the therapeutic culture and contagion from other patients push BID into the open. Though the cultural shaping of the treatment context is inevitable, the absence of a clear boundary between education and indoctrination is relevant in anorexia nervosa, particularly in terms of the patient's age, given that in the case of “early onset” or “onset in infancy” patients lack the possibility of critically examining the therapy rationale. In this sense, the term *indoctrination* is not used here in a pejorative sense but to highlight the active role of mental health professionals.

This is well-documented in Thomas et al.'s (137) report on the ultimate acknowledgment of BID in a congenitally blind patient with anorexia nervosa after her third admission to an Eating Disorders Center. The authors noted: “*Even when directly queried about shape and weight concerns during these first two treatment episodes (e.g., during individual therapy sessions and during body image therapy groups), Ms. A minimized their relevance to her long-standing pattern of restrictive eating. It was not until her third admission that Ms. A began to endorse body image disturbance actively*” (p. 17). Moreover, the authors acknowledged that “*It cannot be ruled out that simply hearing other patients talk about their own poor body image or hearing clinicians target body image concerns in both group and individual therapy may have contributed to the initial development or increased salience of such concerns as legitimate rationales for food refusal*” (p. 18). Nonetheless, Thomas et al. (137) concluded that Ms A. “*exhibited such great preoccupation with body image*,” which was contrary to previous case reports (138–141) suggesting body image concerns may not be central to eating pathology among blind individuals. In the same vein, in Yager et al.'s (142) report of the first case of AN in a blind woman, treatment context contagion was mentioned again by the authors “*Psychiatric admission with other anorexic patients preceded the illness*” (p. 506).

More “spontaneous” migrations toward the typical presentation of AN in North America were reported by Woodside and Twose (143) in the following excerpt: “*The most interesting clinical observation that we could add here relates to the effect of placing such individuals in a group therapy treatment program primarily attended by women with classical North American anorexia nervosa. The inevitable outcome seems to be that over the course of 2 months the women of Chinese or Indian origin acculturate to the dominant culture of the program, gradually abandoning their original rationale for food avoidance and developing a fear of fatness, a drive for thinness, and body image distortion! It is of course not certain that this represents an improvement, although it does allow for a more homogeneous group*” (p. 13).

It appears that in the Eating Disorders Program at the Toronto General Hospital what was typical was not just patients displaying the classical *North American* AN, but also the widespread North American culture inspiring the BID rationale in the treatment of AN patients.

Moreover, white Anglo-Saxon women also experience the acculturation process, as one patient explained: “*I'm still not entirely sure why that is and I could be way off the mark, but I suspect that having significant chunks of my treatment revolve around body image, even when it wasn't currently an issue, may have partly contributed to that faulty line of thought*.” This was written by a former AN patient in a blog (Science of Eating Disorders^22^) as a commentary on Ngai et al.'s (144) paper on the variability of phenomenology in AN. In this paper the authors described four cases presenting all possible combinations where the atypical NFP presentation was consistently present (case 1), evolved from (case 2) or toward FP typical AN (case 4), or was absent in a fourth case representing typical FP-AN (case 1).

Ngai et al. (144) quoted Bryant-Waugh and Kaminski's (145) remark that among the 8- to 14-year-old age-group of “early onset” or “Childhood onset,” the fact that fat phobia emerges as treatment progresses confirms for these authors the denial of this fear early in the illness. However, it is equally valid to view this emergence as a disclosure subject to demand characteristics.

Zanker (146) was even more critical about the pressure to share the preferred language of treatments she received to rescue her from AN: “*In my 20s and early 30s I was unfortunate to experience the embarrassment and humiliation of ‘body image’ therapies. I possessed sufficient insight to recognize that my AN was neither caused nor sustained by distorted ‘body image’, body dissatisfaction or the desire for a very thin body. I did not restrict food and exercise in an obsessive, ritualistic way because I was trying to correct my physical appearance*” (p. 326).

This acculturation process, or perhaps the term *indoctrination* would be more appropriate, appears to have been operative for decades. Thus, Crisp (136) commented on the denial of weight and shape or volume phobia, the overriding terror of fatness of AN patients, and how “o*ften it remains concealed for years and may never be revealed. Only after recovery can anorectics talk more freely about the experiential forces that have been at work in their erstwhile 'illness'*” (p. 687).

Selvini-Palazzoli (32) gave a hint as to the process of turning awkwardness into confidence when she asserted: “*Now it is a well-known fact that anorexics rarely if ever tell the whole truth, except after prolonged and positive psychotherapeutic contacts*” (p. 11). Actually, in the Italian original version of her book there is a final appendix with a table showing demographic and clinical information of the 26 patients she had treated. In 14 of these cases, the duration of psychotherapy varied between 26 and 390 h, and in one case Selvini-Palazoli reported that she had lived with a patient for 4 months.

With regards to denial, determining the amount of pressure that may be reasonably exerted during treatment is a crucial question. In this sense, the case of Lucy, reported by Bruch (147), where at the end of treatment the patient recognized that she had hidden her fat phobia, is paradigmatic. Bruch was probably right^23^, and Lucy hid her fears at the beginning of treatment, but as Thomas et al. pointed out (137) the risk is to try to impose treatment justification on patients who actually lack such fears. In this case, the therapist's insistence could lead to the patient deserting, or at least to a weakening in the therapeutic relationship due to discrepancies in the objectives between patient and therapist. The latter scenario was commented by a former AN patient on the Science of Eating Disorders blog. The blogger criticized the findings of two studies in which the evidence of the absence of BID was not considered to be a lack of BID evidence, but evidence of patient denial. In the first case the study by Gailledrat et al. (148) reported, “*a significant proportion of patients seeking treatment for ED had no or only mild concerns with body shape. This could be of surprise as body shape concerns are core symptoms of ED*” (p. 7). However, the authors rule out this finding because denial “*represents the main bias of this type of study and more specifically in patients suffering from AN-R*.” In the second study, Pilecki et al. (149) explored eating disorders in Poland in the context of Westernization and found that the “thin ideal” score items were “*considerably lower for patients with AN-R than for the healthy control group*.”

Instead of acknowledging these results, the authors were again skeptical about the veracity of patient self-reports. As the blogger commenting on these paper stated: “*in both these studies there is a clear issue with invalidation: some of the participants have indicated that body shape concerns or ‘thin ideal’ pressures are NOT driving their ED – and researchers have refused to believe them. In both studies references are made to authors such as Hilde Bruch or to the DSM definitions to back up claims that the respondents are in denial. They appear to take it for granted that if a participant does not endorse a prevailing social construct theory it is the person living with an ED who is suspect, not the theory. If they are unwilling to accept their participants' responses, you have to question what is the point of even carrying out the research*” (Science of Eating Disorders blog). However, the caution voiced by Russell (12) seems to have been largely ignored, a violation of the wise age-old adage “if the facts don't fit the theory, change the theory,” as it's easier to change the facts, or invalidating them due to the lack of witness credibility.

## Applying Occam's Razor to Psychosocial an Treatments

As we have shown, patient acculturation as mentioned by Woodside and Twose (143) seems to make no difference in terms of patient improvement, although acculturation allows for a more homogeneous group. Furthermore, the patient's resistance to the acculturation process is rated with terms such as denial, active concealment, manipulation, and lying without enough self-criticism of the invalidation that acculturation entails. In any case, acculturation through treatment would be somewhat justified if this process increased the efficacy of the treatment. However, current evidence regarding the efficacy of psychological treatments in AN suggests that this is not the case.

As previously mentioned, SE-AN status refers to the inability of existing treatments to influence the course of the disorder. The space limitations prevent us from reviewing in detail the possible underlying causes of the absence of effective AN treatments [reviewed elsewhere in (150, 151)]. Table 2 shows four sketches of AN treatment representing two “ancient” proposals and two more “modern” treatment proposals.

**Table 2:** Excerpts showing the evolution of Treatment of Anorexia Nervosa across time.

**Déjerine and Glauckler (43)**	**Venables (152)**
We do not hesitate to say emphatically that it is impossible to treat mental anorexia in the family circle, and that to attempt it is to run the risk of certain failure of which the patient's death may be the outcome… Therefore, imperative, and in such cases it must be strict isolation. The desire to shorten its duration may sometimes of itself be enough to induce the patient to consent all the sooner to take food. As far as the alimentation itself is concerned, … Among patients who are still vigorous, as are the majority of the primary anorexias, one manages in 3 or 4 days to get to the point where one can give the classic amount to what constitutes overfeeding in a milk diet. If necessary, -that is, if the patient refuses to take the quantity of milk which is prescribed -one should proceed energetically. One may threaten the patient with the feeding-tube, and if necessary, use it. If he makes himself vomit afterward, as often happens, one must simply begin the gavage over again as soon as he is through. The very important thing is not to give in. As a matter of fact, however, when the physician's authority is sufficiently well established, it is very seldom that one is obliged to have recourse to such extreme measures, because, when he feels that he has to do with somebody who is stronger than himself, the patient generally submits. It may happen that, among certain patients who are extremely weak, one is obliged to seek for aid from ordinary medical therapy; one may thus have to give injections of serum, or hypodermics of caffeine, or camphor oil, to warm the patient by artificial means. These are urgent therapeutic measures such as are applied to people in the last stages of starvation and subjects who are at the point of death… In such patients psychotherapy must not be omitted at the start if the patients are strong enough, or if they have passed the most serious point in the disease where the danger of an unfortunate outcome has been avoided; it is necessary then to try to find out, in the different ways that we have indicated, the emotional, moral, or psychical causes of the anorexic conditions. We do not insist on this point… If the patients have really understood the mechanism of their disease, which at some time you must have explained to them, if you have succeeded by an emotional reaction in penetrating sufficiently into their mentality, it is rare if they do not rapidly comprehend your point of view. At first with effort, but later quite naturally, they will eat heartily and in sufficient quantities. Under these conditions one has no need to fear a relapse. It would, however, be almost fatal if after having made your patient gain a certain number of pounds you should leave him without having modified his mentality (pp. 321−322).	Every patient with anorexia nervosa can be persuaded to eat normally. The condition is hysterical, and the diagnosis of hysteria is only justified when the patient is subsequently cured. No patient should remain uncured, and most certainly no patient should be allowed to die. Directly the diagnosis has been made, treatment should be started. A full explanation should be given to the patient as to the condition present. It is useless to ridicule the sensations of which the patient complains. They are real. Discomfort, distension, repugnance to food and nausea-all are real. The patient has become hypersensitive to abdominal sensation. This is explained, and at the same time it is pointed out that in spite of the patient's sensation he can nevertheless eat, and his stomach, which is normal; can accommodate the food as it is of normal size. Anyone starting to treat a case of this type must be prepared to sit for almost any length of time over a meal. He must never acknowledge defeat and he must never lose his temper. The opportunities for annoyance will be many. He must remember that pure obstinacy is the exception; the patient has a real aversion to food in any form. He must be prepared to fight over every mouthful. With each successive meal taken the task generally becomes easier, and it seldom takes more than a week to establish a normal habit, when the patient can be left to take his meals without the presence of the doctor. From the first a full diet must be taken no matter how insufficient the diet has been in preceding months. Psychological causes should never be discussed at first, unless they are so obvious that they cannot be ignored. Even then I should only be in favor of' pointing out to the patient that I fully recognize that there is a cause for the abstention from food, but that the cause is psychological. It will be found that after the symptom of anorexia has been cured without loss of temper on the part of the physician, the patient's confidence has been gained. Then, if necessary, psychological causes become much more readily accessible and discussion becomes fruitful… I am never in favor of prolonged and deep analysis. The immediate cause is seldom hard to find, and a few frank discussions coupled with any necessary adjustment of circumstances should be all that is necessary. This method has been adopted in all the cases described and none have relapsed, nor have other psycho-neurotic symptoms supervened… All cases should be kept in bed, and it is generally necessary for the patient to have a special nurse. Treatment in the patient's home is most undesirable, and anxious parents should be excluded (pp. 225–226).
**Kidd and Wood** **(****31****)**	**Attia and Walsh** **(****153****)**
The most successful treatment regime appears to be the joint use of insulin and chlorpromazine or reserpine, given in combination with detailed attention to the patient's nutritional requirements and psychological needs. This regime was described by Dally et al. (154) and by Davison and Nabney (155) and its superiority over other forms of treatment was evidenced first by Dally and Sargant (129). This treatment programme is followed at Aberdeen, as in many other centers in Britain and abroad, with encouraging results. Each patient is confined to bed so that energy is conserved, observation is possible and food intake can be supervised. Chlorpromazine is prescribed in increasing dosage according to tolerance. Soluble insulin is given by intramuscular injection in increasing dosage and the resultant hypoglycemia is terminated when signs of sweating and drowsiness appear. Anabolics, vitamins, and high calorie food supplements are also given. Intubation is avoided as it is psychologically undesirable and rarely required. Throughout the early stages of treatment supportive psychotherapy is employed to provide encouragement, an atmosphere of understanding and to mold the basis of the doctor/patient therapeutic relationship. As the patient improves the drugs are reduced, graduated exercise is allowed and psychotherapy is employed to aid the patient in making the necessary readjustments in her psychological functioning that will both militate against relapse and favor better personality and social integration. Mayer (29) has emphasized rightly that psychotherapy should be directed toward specific aspects of the patient's abnormal emotional characteristics. He has described this as a re-educative procedure whereby the patient must gain the insight to learn, first, “to see herself as others see her, as an abnormally and unaesthetically thin individual”; second, to feel hungry and to want to react to hunger by desiring food; and third, to feel fatigue as others do. In short, psychotherapy here aims to reverse the characteristic triad of denfals. In most cases, psychotherapeutic support or management is required for some time after the patient has fully regained normal nutritional and menstrual functioning (p. 447)	Structured behavioral programs generally use a multidisciplinary treatment team that works to deliver consistently applied interventions that aim to normalize weight and eating behavior. Patients are presented with behavioral expectations, such as eating 100% of their food and achieving a significant rate of weight gain. Priority is given to providing patients with adequate calories to begin weight restoration. Approximately 3,500 kcal over maintenance requirements are required for every pound gained. The eating-disorders program at our institution begins with a prescription of 1,800 kcal per day in solid food. When the patient is medically stable (generally, between 2 and 7 days after admission), the diet is increased by 400 kcal per day every 48–72 h until it reaches 3,800 kcal per day, almost all of which (3,000 kcal) is provided in the form of solid food, with the remainder being provided as a liquid supplement. Patients are weighed three times per week and are expected to gain at least 0.75 lb (0.3 kg) over their previous highest weight each Monday, Wednesday, and Friday morning. An important component of the behavioral approach is that contingencies are established in the event that nutritional goals are not achieved. For example, the level of physical activity that is permitted may be decreased or the amount of supervision increased if a patient's weight does not increase by the recommended amount. Calories may be added (including nasogastric feeding) if weight gain is inadequate. Programs emphasize that such interventions are medically necessary to achieve agreed-upon treatment goals, not punishment for “bad behavior.” Opportunities for activity and flexibility within the treatment structure are increased if the expectations are met and reduced if they are not met. Patients participating in behavioral management programs also receive a range of therapeutic interventions from a multidisciplinary team, which may include physicians, psychologists, nurses, nutritionists, clinical social workers, and occupational or recreational therapists. The specific goals of the treatment program include interruption of disordered eating behavior, the reintroduction of “feared” foods, and (especially as normal weight is approached) the practice of normal eating.

As for the first two treatments proposed by Déjerine and Glauckler (43) and Venables (152), both are unsophisticated as compared to the later treatment sketches of Kidd and Wood (31) and the more recent proposal of Attia and Walsh (153). However, there are some common aspects in the treatments proposed by Déjerine and Glauckler (43) and Venables (152); both advocate treatment outside the family^24^ with an emphasis on patient education, and there are no excessive details regarding renutrition. Though both treatments insisted physicians should not give up, there are certain differences in the attitude toward the patient, from a firm and energetic attitude including threats in the case of Déjerine and Glauckler to a more patient attitude in the case of Venables (see Table 2, “*must be prepared to sit for almost any length of time over a meal …, never acknowledge defeat, and must never lose their temper*”). Psychotherapy is secondary, and prolonged and deep analysis is discouraged by Venables^25^.

A radical change in AN treatment profile emerges in the 1960s, as described in the excerpts of Kidd and Wood (31). The combination of insulin and chlorpromazine was popularized by staff at the Department of Psychological Medicine at St. Thomas's Hospital, London (154). One of its members, William Sargant, reluctant to continue practicing leukotomies on patients, himself modified the procedure (158), aware of insulin failure at high doses for restoring patient weight “*to have been singularly unsuccessful in most patients, despite the hunger-producing effects of the insulin regime*” (p. 633), and proposed the combination of insulin and chlorpromazine, also at exceedingly high doses (up to 1,000 mg/day). In a later publication, Dally and Sargant (129) reported the supposed beneficial effects of this combination on body weight by reducing hospital stay and relapse after being discharged from hospital. However, these authors recognize themselves in a subsequent report (159), such promising treatment, which also included supportive psychotherapy and bed rest to avoid possible fractures due to hypotension episodes, did not produce a better outcome during follow-up with respect to a group of patients not receiving chlorpromazine, although it caused serious extrapyramidal effects in up to 50% of cases. Since then, there is no antipsychotic, either typical or atypical, that has not been tested in AN. However, the profuse contumacy in the dozens of clinical trials is only comparable to its inability to rule out the null hypothesis, not to mention weakness in its justification in AN.

A comparison of the two oldest treatments with the two most recent ones underscores both the complexification of treatment (31) and the multidisciplinary recruitment of different professionals (153). However, despite the lack of systematic follow-up studies, as far as the literature can be traced, there are no substantial modifications in the outcome of patients with AN since the 1960s. Thus, one of the preeminent studies published in the 1950s involving a 5-year follow-up of 25 of 38 patients concluded that “*Treatment is unsatisfactory*” [(44), p. 428]. The paragraph summarizing treatment is particularly worth quoting: “*The orthodox treatment, by supervised diet, was the one most often used, and was employed at some stage in 60% of the patients. Modified insulin was frequently used as an adjunct to dietary methods. Endocrine preparations of various kinds were given in 30%. Including therapy given in other hospitals, 25% (9 patients) received E.C.T., leucotomy, or insulin shock. 2 of the 3 patients who had leucotomy benefited, but the follow-up periods do not exceed three years. If, as we believe, most of these patients have personality difficulties, good long-term results following leucotomy are not to be expected. One patient gained a stone during a course of E.C.T. In another case, no improvement occurred after 15 deep insulin comas. A few had no treatment, because they either died, or else recovered, too soon. 30% received some form of psychotherapy, excluding simple reassurance and persuasion*” [(160), p. 671, underlined was italics in the original]. In hindsight, it cannot be ruled out that this type of clinical management had significant iatrogenic effects in patients receiving such treatment^26^.

Interestingly, and despite their poor results, Kay and Leigh (44) declared that “*The time-honored treatment, by persuasion and meticulous supervision of the patient's diet, practized so success fully by earlier physicians, has in recent reports been found less satisfactory*” (p. 426). What had changed in the space of 20 years for the treatment proposed for example by Venables (152) to be no longer satisfactory? Perhaps the priced payed for the growing specialization was that patience, tact, and gentle understanding succumbed to the maelstrom of advances in technology and resources (antipsychotics, insulin, ECT, leucotomy, and so on) that left in the background essential elements of the doctor-patient relationship, as pointed out by Magendantz and Proger (15): “While *endocrine substitution therapy is being pushed, one should not forget the probable great importance of patience, tact and kind understanding of the patient's personal problems, a therapeutic approach in which the older type of physician seems to have been superior to our highly specialized, modern physician*” (p. 1,983)^27^.

Paralleling the absence of efficacy of pharmacotherapy (162–164) the state-of-the-art regarding current evidence-based treatments after several randomized control trials barely exceeds a general “positive care effect”^28^ (165, 166). Currently, a “tie score effect” summarizes evidence gathered from comparative efficacy studies as no treatment has shown to be clearly superior to any other. In addition, this result applies to the treatment known as Specialist Supportive Clinical Management [SSCM; (167)]. However, SSCM is a rebranding of a treatment previously referred to as Non-specific Supportive Clinical Management (NSCM), used as an active control condition in randomized controlled trials for AN that ended up being considered an effective therapy in its own right (151). On account of its efficacy, similar to other specialized brand type treatments (enhance cognitive behavior therapy—CBT-E, psychodynamic—PSYCH, interpersonal—ITP), the original authors justified their name change, and recently outlined the properties of SCSCM, despite not being based on any “*theoretical model of causation or theory-driven strategies*” (168). However, despite this apparent shortcoming, SSCM was effective instilling hope (169), and it was indistinguishable from Cognitive Behavioral Therapy for AN (CBT-AN) as “*both treatments were able to promote moderate therapeutic alliance in early treatment, increasing to strong therapeutic alliance in late treatment, to relatively the same degree*” [(170), p. 787].

McIntosh et al.'s (171) study was relevant for an additional reason to the appearance on the scene of SSCM treatment, which has since become the quintessential comparison treatment. Although originally conceived as a non-specific treatment, SSCM worked just as well as the other two treatments, CBT and ITP. This meant, for example, that the focus on weight and shape concerns, a central element of CBT, and the BID construct, made no difference in the outcome. Moreover, the same conclusion holds with respect to the interpersonal assumptions derived from treatments developed for other disorders such as depression, as in the case of ITP.

Moreover, subsequent evidence on the parity of SSCM with respect to two more treatments, the Maudsley model of anorexia nervosa treatment for adults (MANTRA) and enhanced-CBT (E-CBT), merit mention. Thus, SSCM fared equally well to MANTRA (172–174), a modular, research derived treatment based on a cognitive-interpersonal maintenance model of AN (175) that according to their designers “*does not emphasize weight and shape concerns, something that may be surprising to some readers*” [(176), p. 346]. SSCM was also found to be equally efficacious as a new treatment founded on a transdiagnostic model of eating disorders (62). This new version of the treatment added to the original cognitive conception of AN as a “disorder of control” (177) four additional maintenance mechanisms, namely clinical perfectionism, mood intolerance, low self-esteem, and interpersonal difficulties.

There is no simple interpretation of the collective results of clinical trials that have included a treatment such as SSCM, which could be considered of “low therapeutic intensity,” compared to conventional psychosocial treatments such as CBT, ITP, MANTRA, and CBT-E. However, it is not far-fetched to say that for both AN in general, and SE-AN in particular, *less is more*, and that when that maxim is not respected, a high drop-out rate is the outcome^29^. Thus, it may also be reasonable to ask to what extent the status of a patient's SE-AN may also depend on the aggressiveness of the acculturation processes inherent to previous treatments. In this sense, SE-AN status is indicative not only of the intrinsic refractoriness due to low motivation of patients with AN but also of the iatrogenic effects of previous treatments. Exposure to treatments that do not respect either the rhythm or the peculiar low motivation of these patients, and focus exclusively on modifying egosyntonic aspects within the precarious equilibrium of the patient, are doomed to failure. The notion of respecting the rhythm of the patient is present in several quotations throughout this paper as manifested by some authors at the beginning of the twentieth century. For example in Table 2 when Venables states, “Anyone starting to treat a case of this type must be prepared to sit for almost any length of time over a meal. He must never acknowledge defeat and he must never lose his temper.” Likewise, Farquharson and Hyland (see text footnote^27^) noted that “the patients can often be helped by kindly support, calm discussion, explanation and persuasion, all entirely free from censure.” In the same vein, we can place the “non-interpretive and fact-seeking approach” recommended by Bruch [(34), p. 336], who advised against any authoritarian attitude in therapy, i.e., avoiding telling patients how they should feel and think, as this would further reinforce their sense of profound ineffectiveness. More recently, in order to strength the cooperation with AN patients, Attia and Walsh (179) emphasized shifting the focus toward the ego-dystonic consequences of the restrictive eating pattern of patients with AN, such as lack of concentration, anxiety, depression, increased irritability, hair loss, or feeling cold.

Finally, there is a disconcerting aspect in the intrinsic nature of AN, the recovery of a significant number of patients not receiving formal treatment. Though SE-AN is a serious chronic disorder, the spontaneous remission of the disorder was first noted by Lasègue himself (79). Likewise, Farquarson and Hyland (37) pointed that “*Sometimes they improve spontaneously with a change from a stressful environment*” (p. 418), and the majority of AN patients with AN nervosa “*recover with relatively simple treatment*” (p. 419). This affirmation was made after a 20- to 30-year follow-up of 15 AN patients treated between 1932 and 1943, where “*With one exception these patients all made good recoveries from their initial illness*” (p. 418).

More recently, the first paragraph opening a report describing factors associated to AN recovery from a Finnish population-based study Keski-Rahkonen et al. (180) reads: “*A substantial proportion* [of AN patients] *attains complete recovery, even without formal treatment, but about one in five suffers from a chronic disorder that carries a high risk of mortality*” (p. 117). The term *substantial* was probably chosen by these authors as they found in their study that “*The 5-year clinical recovery rates were similar for the detected and undetected cases: 61.8% versus 68.4%, respectively, for DSM-IV anorexia nervosa, and 60.1% versus 69.5%, for broad anorexia nervosa*” [(181), p. 1,263]^30^. However, a shortcoming of this study is its retrospective nature (182).

Moreover, in an up-to-date unique controlled, 30-year follow-up study of adolescent onset AN, the Gothenburg anorexia nervosa study (134), the authors reported “*one in four people had never received treatment for an eating disorder. Nonetheless, treatment did not affect the outcome 30 years after the onset of anorexia nervosa*” (p. 5). This finding corroborated a study in Adelaide, Australia, “*where many patients make a good recovery without accessing to specialized treatments of any kind*.” In fact, receiving treatment^31^ “*did not alter long-term outcome either as predicted by variables gathered at recruitment, or by combination of initial and 6-month variables*” [(183), p. 1,256].

## Conclusion

As highlighted in the first section of this paper, interference from the clinician's theoretical framework is easily identified in psychoanalytic accounts predating the appearance of the BID construct. This interference is less obvious now that BID is considered a core element in AN diagnosis. In fact, difficulties in getting the patients' acquiescence of BID symptoms required to fulfill diagnostic criteria are conventionally explained as conscious tricks or negations to mislead well-intentioned busy clinicians. However, given the obvious asymmetries in age, power status, and context familiarity between an adolescent and an adult doctor in an unfamiliar hospital ward, or an eating disorders unit, there should be greater awareness about the impact of such asymmetries.

The perpetuation of the symptomatic profile for AN from *DSM-III* to present *DSM-5* gives clinicians the deceptive reassurance that the criteria for AN diagnosis grasp the essence of the disorder, ignoring the issues we have previously addressed (invalidation, acculturation, and indoctrination). It is easy to agree that at a behavioral level AN patients act “as if” they were deeply invested in a relentless pursuit of thinness. Historically, the egosyntonicity of weight loss in AN patients has been documented, as aptly reflected by the German term *magersucht* for anorexia nervosa. However, this commitment to thinness has been equated to other allegedly synonymous actions that go beyond simple avoidance to putting on weight such as weight phobia, fat phobia, or intense fear of gaining weight or becoming fat, as stated by Rieger et al. (184). Thus, from the point of view of BID culture, it is reasonable to view the act of diagnosis itself as the beginning of a process of acculturation and even indoctrination.

We have already seen how difficult it is to justify the lack of clinical expertise by most clinicians from Gull and Lasègue before the advent of Hilde Bruch's proposals regarding the characterization of typical AN in terms of BID construct. On the other hand, we have also reviewed how the current diagnostic criteria that nowadays define a typical AN picture were absent in patients during a period of time (1860/1873–1962) much greater than that elapsed since the original BID formulation until today (1962–2020). Similarly, current conception of typical AN conveys the suspicion of denial and concealment in cases where the patient experience does not conform to the report expected by doctors and researchers. This atmosphere of pressing acculturation is as unrecognized as the responsibility of clinicians and researchers spreading it widely through scientific literature and mass media and thus approaching susceptible people like Russell pointed out (12). Seen from this perspective, bad outcome of SE-AN could result from the interaction between disoriented underweight adolescents who can't help themselves to stop their restrictive eating and excessive activity on one side and ineffective treatments based in rationales that impose a patient's acquiescence of the BID construct on the other side.

Likewise, with hindsight, it is easy to consider that some of the treatments in the past considered the best treatment available (see Table 2) were unaware of iatrogenic effects (185)^32^. Evidently, the same shortcoming may be said of the theoretical assumptions underpinning such treatments. In both cases, if we apply the definition of chronicity that opens this document, it would be clear that chronicity would have been the result of flagrantly ineffective treatments and faulty assumptions.

As for the state of the art of current treatments, the qualifier “evidence-based” means weak evidence as we are still far from reaching the criterion of strong evidence. The “Dodo Bird” verdict defining the current landscape of psychosocial treatments in AN depicts our repeated failures in the development of an effective treatment for AN (186). We should not discard the possibility that the SE-AN category may be echoing that we have overlooked the Hippocratic dictum *Primum non nocere*. Only time will probably give us the answer. In the meantime, as Freeman Dyson encouraged (187), we should not forget the scientific mandate that any prevailing dogma, as in the case of the BID construct, must be challenged.

## Author Contributions

Both authors made equal contributions to the manuscript and approved it for publication.

## Conflict of Interest

The authors declare that the research was conducted in the absence of any commercial or financial relationships that could be construed as a potential conflict of interest.

## Footnotes

^1^In this paper the acronym BID is used to refer to the body image disturbance as a generic construct, much the same as Treasure et al. (8) who listed under the umbrella of body image disturbances “*Weight and shape concerns (e.g., preoccupation with weight, shape, or both); Overvaluation of shape and weight in determination of self-worth; Minimisation or denial of symptom severity; Disturbance in the way body is experienced [and] Intense fear of weight gain, even though underweight*” (p. 584). Other authors, i.e., Fairburn and Brownell (9), group together “*drive for thinness, fear of fatness, shape and weight dissatisfaction, body size misperception, body image disturbance, and fear associated with physical maturity*” (p. 143), as different ways to describe the psychopathology related to weight and shape.

^2^According to data supplied to him by Peter Slade, the 165 publications in 1982 almost tripled as compared to the previous 59 references in 1971. The corresponding numbers for the same search in Pubmed for 2019 yielded more than a thousand references.

^3^An interesting example of the interference of the observer's assumptions was provided by Moulton (17) where her description of the discomfort displayed by an adolescent during nasogastric intubation was seen through the glass of the oral impregnation fantasies theoretical frame: “*For the first five months in the hospital, she was tube-fed daily by different doctors. She said that ‘only daddy can put it down without hurting,’ and took it with passive disgust, meanwhile making obviously erotic pelvic movements*” (p. 72).

^4^Ten years later Moulton (17) interpreted the discomfort displayed by an adolescent during nasogastric intubation through the lens of the oral impregnation fantasies theoretical frame: “*For the first five months in the hospital, she was tube-fed daily by different doctors. She said that ‘only daddy can put it down without hurting,’ and took it with passive disgust, meanwhile making obviously erotic pelvic movements*” (p. 72).

^5^Within the psychoanalytic formulation, the fantasies of pregnancy were nothing more than the elaboration of the difficulties of adolescents in their transition from adolescence to young adulthood as Rank (22) had pointed out: “*The separation of the growing individual from parental authority it is one of the most necessary achievements, but also one of the most painful of evolution*” (pp. 63–64). In fact Loeb (23) proposed useful “*to conceive of this 'Peter Pan' syndrome as the acting-out of the wish not to grow, to remain always the favored dependent child, unthreatened by the demands of sexuality, of adulthood with its attendant responsibilities*” (p. 450).

^6^She defined adultomorphizing as the “*tendency of interpreting unknown behavior in terms of adult meaning that psychoanalysts have tacitly accepted from Freud*” [(26), p. 3].

^7^Mayer (29) is one of the rare cases that is skeptical of Bruch's third characteristic of a sense of profound ineffectiveness in AN patients; in his experience Mayer stated he “*has not received this impression from the patients he has seen*” (p. 531).

^8^In a posterior paper Bruch (30) introduced certain inconsistencies in stating that the sample of 12 patients included those treated between 1955 and 1959, half the time period cited in the 1962 paper: “I saw only six cases between 1942 and 1954, 12 from 1955 to 1059, and 25 from 1960 to the middle of 1964” [(30), p. 556].

^9^Selvini Palazzoli (32) also added the synonymous essential “*anoressia mentale essenziale*” (p. 29), and more recently Strober (33) still added two more synonyms, *pure* and *prototypic*.

^10^It is interesting to note that Cappon quoted not Bruch's (28) original work, but a paper read at the first international symposium on anorexia nervosa in Gottingen in April 1965. Nevertheless, just a few months later, Cappon judged BID was already cardinal.

^11^This list of initiating factors was quite similar to that annotated by Déjerine and Glauckler (43) in cases of true or primary mental anorexia such as “*Grief, disappointment in love, or unhappiness in marriage are very often the emotional source of the most characteristic mental anorexias*.” *In other cases the restriction is at first voluntary and intentional. It is often due to coquetry* … [Dejerine and Glauckler also list] mysticism and poverty where persons “*obliged by the necessities of life to deprive themselves to such a degree that, when the illness or lack of employment which has caused these privations has disappeared, they find it impossible to take food again*” (pp. 4–5).

^12^In addition, Selvini Palazzoli (32) underlined in primary AN “*la necessita di divenire emaciate*” (p. 32), which was over translated into English (32) as a “*desperate need to grow thinner*” (p. 23), perhaps to parallel Bruch's description of primary AN as a relentless pursuit of thinness.

^13^Relevant to Bruch's third characteristic of a sense of profound ineffectiveness in AN patients is the comment on the young men participating in the Minnesota starvation study, “*They became discouraged because of their relative ineffectiveness in daily living. Inability to sustain mental or physical effort contributed much to this feeling of inefficiency*” (p. 33).

^14^Although Bruch (34) acknowledged in her book *Eating Disorders: Obesity, Anorexia Nervosa, and the Person Within*, the characterization of AN in terms of “weight phobia” made by Crisp (93), or the “morbid fear of fatness” by Russell (94), are contributions she could have claimed as her own given that in her 1966 paper she fully addressed the distinction between typical and atypical AN: “*It is of decisive significance whether a patient is preoccupied with its body size, with a relentless pursuit of being thin and a phobic avoidance of being fat*” [(30), p. 555].

^15^In the pre-*DSM* era Crisp (99) made a similar reasoning when he wrote: “*If the physician docs not have the disorder of weight in mind when he is interviewing the patient, he may misdiagnose the patient as having a neurotic adolescent disorder only reflecting whichever of the above behavior patterns and mood states is most evident at the time*” (p. 494).

^16^Janet describes how Charcot, “*while undressing a patient of this kind, he found that the adolescent wore on her skin, fastened very tight around their waist, a rose-colored ribbon. He obtained the following confidence; the ribbon was a measure which the waist was not to exceed. ‘I prefer dying of hunger to becoming big as mamma*” [(80), p. 234].

^17^The atypical group is not a homogeneous category according to a recent study conducted in Japan (110). In this study, significant differences were found between a group of patients (NFP-AN) who did not meet Criterion B or Criterion C of the *DSM-IV* criteria, and the AN-NED group not fulfilling criterion C.

^18^This active role could be an explanation for the unprecedented change in the proportion of typical FP-AN vs. atypical NFP-AN in Hong Kong. While a ratio around 40/60 was documented in the first reports in the first period 1987–1989, in the 2005–2007 period, the proportion had changed to 20/80 (117). How was this change possible?

^19^This change implies a distancing from the position held by Bruch, for whom “*This absolute denial of their starved appearance is pathognomic for primary anorexia nervosa*” [(122), p. 216].

^20^Lack of concern regarding its cachexic appearance has been a feature repeatedly documented in the literature. It was remarked by Berkman, “*they do not express concern over their loss of weight or cachetic appearance*” [(40), p. 681], or by Nemiah, “*especially striking is the lack of concern the patient shows for the seriousness of her physical condition and for her often shocking appearance*” [(25), p. 259]. Recently, a lack of concern and behavioral activation have been postulated as core symptoms of anorexia nervosa (42).

^21^In the same vein, the late Crisp (123) also excused Gull and Lasègue for not having been able to glimpse the fat phobia in their first description of AN disorder because: “*Refusal, in the initial clinical consultation, to recognize or admit to any phobia of normal adult body weight is common enough, especially if the history of initial pursuit of weight reduction has been kept a secret. It is unlikely to have come readily to the attention of those recognizing and struggling to help people with the disorder in the 19th century, when it was first labeled*” (p. 197).

^22^https://www.scienceofeds.org (accessed February 4, 2020).

^23^As reported by Bruch (147), Lucy confessed: “*It is so easy to say you are ‘full’–it is a lie- it is only a justification. You have to deny yourself food. You can't say ‘I want to be skinny,’ not really, so you have to come up with a reason for everybody else. So you say you are full. It is easy to say ‘I can't eat that much’*” (p. 81).

^24^Although Lasègue (79) is generally considered first advocating isolation of patients from his parents (“worst attendants” according to Gull) it was Marcé (10) who stressed this parentectomy in the two cases described in his seminal work on AN.

^25^This recommendation against “*elaborate psychotherapy*” [(156), p. 746], or “*elaborate psychiatric methods*” [(39), p. 737], as well as the most blunt: “*in severe cases of anorexia nervosa it seemed to him almost criminal to waste time in anything like psychoanalytic investigations (however interesting they might be) at the commencement of treatment*” [(157), p. 745] can be found in the discussion following a conference presented on January 24, 1939, by Ryle (39).

^26^For patients receiving this treatment Kay (160) reported a recovery limited to 10–20% of the sample. However, regarding treatments Kay stated: “*As far as we know, the course of the disease is not influenced by endocrine therapy; psychotherapy has given poor results, as have other treatment methods*” (p. 674). It should be noted that this percentage was significantly lower than reported by Theander (91) at six-year follow-up for patients admitted to somatic and psychiatric clinics in southern Sweden for the period 1930–1960, with a recovery rate of 55%.

^27^In the line of the soft treatment exemplified in the excerpt from Venables (152) included in Table 2, and the caution concerning deep psychoanalytic treatment, Farquharson and Hyland (161) state that “*Attempts to modify the abnormal attitude by intensive psychotherapy or by psychoanalysis has usually proved ineffective and sometimes worsens the condition. Fortunately, the patients can often be helped by kindly support, calm discussion, explanation and persuasion, all entirely free from censure*” (p. 418).

^28^A general positive effect in which the improvement might not be due to the treatment itself since the effect of spontaneous recovery should be taken into account, as mentioned by Theander (91) 50 years ago in his follow-up study of patients with AN when he said: “*Therefore, in the evaluation of a therapeutic method there is always reason to ask: to what extent are the results reasonably due to the treatment, and to what extent to 'vis medicatrix naturae'. The natural course of the disease should thus always be considered*” (p. 115, underlined was italics in the original).

^29^In this respect, in Touyz et al.'s (178) study the recalibration of aims in both treatments (SSCM and CBT-AN) corresponded with a low dropout rate of SE-AN patients.

^30^Undetected cases amounted for 47% of all *DSM-IV* AN cases, and 59% of all broad AN cases. Cases undetected by the health care system were those never receiving an eating disorder diagnosis from a health professional whereas the other cases were detected by the health care system, although this detection didn't imply they had received treatment for their disorder.

^31^Treatment was categorized according to modality and intensity: “(1) no treatment; (2) minimum treatment (one to three sessions of outpatient treatment after assessment, with no other secondary treatment); (4) extended outpatient treatment (greater than three sessions with any kind of specialist mental health professional, but no inpatient care); (5) short inpatient treatment (<2weeks' inpatient treatment for any mental health disorder, including an eating disorder); and (6) extended inpatient care (>2 weeks' admission for an eating disorder)” (p. 1,254).

^32^Iatrogenic effects due to the etiological uncertainties were sadly mentioned by Magendantz and Proger (15): “*the practical handling of these cases has been difficult and has varied from psychoanalysis to risky, repeated implants of animal pituitary tissue into the omentum of these patients of low resistance. How many of these unfortunate girls have been sent to tuberculosis sanatoriums as was our first patient, who at the outset had no evidence of pulmonary tuberculosis? How many have died in psychiatric institutions classified as schizophrenic or depressive?*” (p. 1,983).
